# Life’s building blocks: the modular path to multiscale complexity

**DOI:** 10.3389/fsysb.2024.1417800

**Published:** 2024-07-17

**Authors:** Saúl Huitzil, Cristián Huepe

**Affiliations:** ^1^ Engineering Sciences and Applied Mathematics, Northwestern University, Evanston, IL, United States; ^2^ Northwestern Institute on Complex Systems, Northwestern University, Evanston, IL, United States; ^3^ CHuepe Labs, Chicago, IL, United States

**Keywords:** modularity, multiscale modeling, hierarchical organization, emergent complexity, evolutionary structures, modular evolution, evolutionary dynamics, biological complexity

## Abstract

Modularity, the structuring of systems into discrete, interconnected units, is a fundamental organizing principle in biology across multiple scales. Recent progress in understanding the role of modularity as an evolutionary mechanism and a key driver of biological complexity has highlighted its importance in shaping the structure and function of living systems. Here, we propose a unifying framework that identifies the potential evolutionary advantages of modularity in systems ranging from molecular networks to ecologies, such as facilitating evolvability, enhancing robustness, improving information flows, and enabling the emergence of higher-level functions. Our analysis reveals the pervasiveness of modularity in living systems and highlights its crucial role in the evolution of multiscale hierarchies of increasing complexity.

## 1 Introduction

Modularity is a fundamental organizing principle in biological systems that manifests itself at multiple scales and levels of organization (Ravasz et al., 2002; Meunier et al., 2009; Lorenz et al., 2011). Although its precise definition can depend on the context, in a broad sense, modularity in biology has been connected to the capacity of living systems to be “near decomposable,” (Simon, 1962), that is, to their ability to divide functions into different subunits known as modules, which perform specific tasks with a certain degree of autonomy (Wagner et al., 2007). These modules can be viewed as composed of parts that interact more closely with each other than with other modules, thus showing a degree of functional independence that allows them to perform specific functions efficiently (Klingenberg et al., 2003; Cheverud et al., 2004; Kadelka et al., 2023). Modularity is also closely related to the emergence of hierarchical organization, in which systems are organized into nested levels, where each level is composed of subsystems from lower levels and, in turn, forms part of supersystems at higher levels (Barabasi and Oltvai, 2004).

Modularity is a multifaceted concept that has been studied through diverse perspectives, including developmental, evolutionary, genetic, and morphological approaches, each with its own set of questions, methods, and insights (Zelditch and Goswami, 2021). For example, network theory provides a quantitative framework for analyzing modularity based on topological features, while other approaches focus on the physical structures found in living organisms, such as the organization of cells into tissues and organs or the arrangement of skeletal elements (Melo et al., 2016; Felice et al., 2018). Other studies have explored the modular functional interactions among components, such as gene regulatory networks and metabolic pathways (Raff, 1996; Wagner et al., 2007). While these approaches have different emphases and may not always fully address the origins, evolution, or implications of modularity, their collective findings highlight the ubiquity of modular organization in biological systems. This suggests the existence of a universal principle driving the emergence of complexity, whereby simpler subsystems agglomerate into stable combinations that become the building blocks of larger and more intricate structures and functions, potentially leading to the formation of hierarchical layers through successive combinations of components and subcomponents (Schaffer and Ideker, 2021). In this context, biological complexity is understood as the degree to which a system comprised of interrelated components can collectively exhibit emergent properties and behaviors that are more than the sum of its parts (Lobo, 2008). To fully understand the role of modularity in the organization of life, an integrative approach that synthesizes insights from different perspectives and considers the origins, evolution, and implications of modularity across multiple scales is necessary.

The fundamental role of modularity in the evolution of biological complexity is evidenced by its presence in a great diversity of living systems (at multiple scales). For example, the modular organization of cells is considered a crucial factor in the emergence of higher life forms. As highlighted by Lynn Margulis’ groundbreaking work on endosymbiotic theory, the origin of eukaryotic cells is a prime example of how modularity has driven the emergence of more complex forms of life (Sagan, 1967; Gray, 2017). According to this theory, the modular integration of specialized organelles (such as mitochondria and chloroplasts), which evolved from symbiotic bacteria, allowed for greater efficiency in cellular processes and played a key role in the appearance of eukaryotic cells (Schliwa and van Blerkom, 1981).

The emergence of multicellularity is another notable example of how modularity has driven the evolution towards increasing complexity, as discussed by Smith and Szathmary (1997) in “The Major Transitions in Evolution.” This seminal work explores the role of modularity in the evolution of life, from the integration of replicating molecules into chromosomes to the origin of societies. Organisms like *Volvox carteri*, which appear to be in a transitional stage towards multicellularity (Kirk, 2005), demonstrate how the organization of cells into modules can give rise to more complex life forms. In more advanced multicellular organisms, modular specialization extends to tissues and organs, thus enabling the emergence of highly complex adaptive systems (Bonner, 1988; Wagner and Altenberg, 1996).

The holobiont concept (increasingly relevant for systems biology) further illustrates how modularity and hierarchical organization enable the emergence of higher levels of complexity in biological systems. The holobiont refers to the collective biological entity formed by a host and its associated microbiome, functioning as an integrated and coherent unit of evolution (Bordenstein and Theis, 2015; Rosenberg and Zilber-Rosenberg, 2018). Just as the modular integration of organelles gave rise to eukaryotic cells, and the modular organization of cells led to multicellular organisms, the holobiont represents a higher level of modular organization, where the host and its microbiome form a collective organism that is more complex and adaptive (Huitzil et al., 2018; 2023). Its hierarchical organization allows for the emergence of novel properties and functions that are not present in the individual components (Huitzil et al., 2020; Huitzil et al., 2023), enabling holobionts to adapt to diverse environments and respond to challenges more effectively than either the host or the microbiome could alone.

At even larger scales, populations and ecosystems also exhibit modular organization, forming complex networks of interactions where groups of species interact more closely with each other than with other groups, (Pimm, 1991; Sole and Montoya, 2001). Moreover, superorganisms, such as bee and ant colonies, represent a further level of organization into modular structures and functions where groups of individuals specialized in different tasks contribute to the efficiency and adaptability of the colony as a whole (Holldobler and Wilson, 2009).

Multiscale modularity is not only a property observed in the structural organization of biological systems but must also have important implications for their evolution and adaptation. For example, modular organization allows for the evolution of new functions through the modification and recombination of existing modules, without disrupting the entire system, while a hierarchy of modules allows for evolution at multiple levels (Simon, 1962; Kashtan and Alon, 2005; Wagner et al., 2007). This flexibility may have been a key factor in generating the great diversity and complexity of life on Earth. Various models and conceptual foundations have been developed to better understand the evolutionary implications of multiscale modularity, which we briefly describe in the next section.

## 2 Models and theoretical foundations

The concept of modularity has been explored from various perspectives to understand its role in the organization and evolution of biological systems across multiple scales. One of the most influential contributions in this field is the work by Simon (1962), who introduced the idea of “nearly decomposable systems” described in the introduction. This seminal work laid the foundations for understanding how hierarchical modularity can facilitate the efficient evolution and adaptation of complex systems by reducing the interactions between subsystems. Building upon these ideas, the study of modularity has been approached from different angles, including network theory, evolutionary biology, and systems biology, to unveil the principles governing the emergence and maintenance of modular organization in living systems.

Further advances in the study of modularity have revealed its crucial role in shaping the structure and function of biological networks. For instance, Ravasz et al. (2002) demonstrated that metabolic networks exhibit a hierarchical modular organization, with highly connected modules composed of smaller, less connected modules. This hierarchical structure was shown to be related to the functional classification of metabolic reactions, suggesting that modularity and hierarchy are essential for the efficient functioning of metabolic systems.

The complexity of biological systems and their modular and hierarchical organization have inspired the development of mathematical and computational models that seek to capture fundamental principles underlying these phenomena. These minimal models have been crucial for understanding how modularity and hierarchy can emerge and evolve in complex adaptive systems (Hartwell et al., 1999; Alon, 2007; Solé and Valverde, 2008). Optimization-based models, in particular, have been instrumental in understanding the evolution of modularity (Kashtan and Alon, 2005; Clune et al., 2013; Mengistu et al., 2016). Kashtan and Alon (2005) demonstrated that modularity can evolve in networks when the environment changes in a modular fashion, suggesting that modularity is an adaptive response to certain features of the environment.

Another important line of theoretical research has focused on the evolutionary mechanisms that give rise to modularity in biological systems. Wagner et al. (2007) reviewed the concept of modularity from an evolutionary perspective, discussing how natural selection can favor the emergence of modular architectures. They argued that modularity enhances evolvability by allowing for the independent evolution of different functional modules, thus enabling the exploration of new adaptive solutions.

Network theory has provided a quantitative framework for analyzing modularity based on connectivity patterns. Models such as the “preferential attachment” model by Barabási and Albert (1999) and the evolving modularity model by Valverde and Solé (2007) have helped to understand how modular architectures can emerge in biological networks. These suggest that modularity can arise as a result of selection for both robustness and evolvability.

Collectively, all these minimal models have provided valuable insights into the mechanisms and principles underlying the emergence and evolution of modularity and hierarchy in biological systems. However, many challenges lie ahead, such as integrating these principles into more realistic modeling frameworks that capture the complexity of biological systems at multiple scales and the empirical validation of these theoretical predictions.

In summary, the theoretical foundations for describing the origins and properties of hierarchical modularity in biological systems have been explored from different perspectives, including complex systems theory, evolutionary biology, and network theory. These efforts have revealed the emergence of modularity at multiple scales as a fundamental organizational principle that can confer key evolutionary advantages to biological systems, such as adaptability, robustness, and efficiency.

## 3 Advantages of modularity

To advance towards a universal theory of the role of modularity in the development of complex life forms, we must first identify the evolutionary advantages (EAs) that this type of structure may provide, regardless of the specific features or scale of the system. By considering various theoretical and experimental realizations of modularity, we propose here a general classification of the key EAs of multiscale modularity into four classes that can be identified in a variety of biological systems. These EAs can be briefly listed as follows:

**EA 1** The reuse and recombination of modular components facilitate the evolution of new functions and rapid adaptation of organisms to changing environments (Patthy, 1999; Bashton and Chothia, 2007).
**EA 2** Modularity enhances the robustness of biological systems by limiting the propagation of perturbations and allowing for the independent evolution of sub-systems (Wagner et al., 2007; Samal and Jain, 2008).
**EA 3** Hierarchical modularity enables the efficient processing and integration of information across multiple scales of biological organization (Barabási et al., 2003; Meunier et al., 2009; Maier et al., 2019).
**EA 4** Modularity enables the integration of simpler components into more complex systems, providing a pathway for the evolution of biological complexity, the division of labor, and the emergence of novel functions (Baldwin and Clark, 2000).

These advantages play a crucial role in the emergence of modular organization across multiple scales. By facilitating adaptability, robustness, efficient information processing, and the integration of simple elements into more complex components, modularity allows for the evolution and survival of increasingly complex living systems. This process can develop iteratively, with modules at one level serving as building blocks for higher-level modules, leading to the formation of multiple nested hierarchies of modular structures at larger and larger scales.

## 4 Biological examples

To illustrate the evolutionary advantages of modularity presented in the previous section, we will briefly describe a series of examples that demonstrate how the key benefits of modularity manifest themselves in concrete biological systems, providing evidence for the central role of modularity in shaping the self-organization of structure and function in living systems.

At the molecular level, the modular architecture of proteins allows for the recombination of functional domains, facilitating the evolution of new functionalities, which corresponds to an advantage of type EA 1. For instance, the shuffling of protein domains through mechanisms such as exon shuffling and gene duplication has been a major driver of protein evolution (Patthy, 1999). This modular organization enables proteins to adapt rapidly to new challenges without the need to evolve entirely new structures from scratch.

Gene regulatory networks provide another example of a type EA 1 benefit of modularity. The lac operon in *E. coli*, for instance, is a modular regulatory system composed of a promoter, an operator, and structural genes that control the expression of enzymes involved in lactose metabolism. This modular structure facilitates the efficient control of gene expressions and has been found to regulate different metabolic processes in other bacterial species, thus showing that it can be reused and adapted to control diverse functions (Browning et al., 2019). Similarly, the eukaryotic cell cycle is regulated by a modular network of interacting proteins (cyclins and cyclin-dependent kinases), with each protein complex forming a functional module that drives a specific phase of the cycle (Schulze-Gahmen et al., 1995). The modular organization of these regulatory networks enables the reuse and recombination of regulatory modules, facilitating the emergence of new functionalities and the adaptation to diverse environmental conditions.

We can also identify the benefits of modularity in the very different context of cognitive processes. In this case, modularity allows the brain to efficiently process complex information by integrating specialized modules that operate in a relatively autonomous manner (Sperber, 2002; Carruthers, 2006), which corresponds to a type EA 3 case. This organization enables the coexistence of functional specialization and integration, as exemplified by language processing, which involves the coordination of multiple specialized modules, such as phonological, syntactic, and semantic processing units (Fodor, 1983; Robbins, 2009). The modular structure of brain networks is hierarchically organized, with smaller, more specialized modules nested within larger, more integrative modules (Meunier et al., 2009). This hierarchical modularity allows for efficient information processing within specialized domains while also enabling the emergence of higher-level cognitive functions through the integration of these modules. It can thus be characterized as conferring not only type EA 3 but also type EA 4 advantages.

In yet a different context, at the ecosystem level, it has been shown that modularity contributes to stability and resilience by compartmentalizing interactions between species, which corresponds to a type EA 2 benefit. In this case, modular ecosystems are characterized by groups of species that interact more strongly within modules than between modules (Olesen et al., 2007). This compartmentalization can limit the spread of perturbations and prevent cascading failures across the entire ecosystem (Stouffer and Bascompte, 2011), thereby enhancing robustness.

Finally, an example of a type EA 4 advantage of modularity can be found in the modular organization of metabolic networks, where the integration of simpler modules allows for the generation of more complex metabolic capabilities. Photosynthesis, for instance, comprises distinct modules, such as light-harvesting complexes and electron transport chains, which integrate to convert light into chemical energy (in the form of ATP and NADPH) (Stirbet et al., 2020). Similarly, the citric acid cycle consists of a modular assembly of enzymatic subunits that form an integrated functional module, which enables the evolution of novel metabolic functions through the recombination of existing modules. In both cases, modularity enables the hierarchical integration of simpler modules into more complex metabolic systems, facilitating the emergence of novel functionalities. For example, photosynthesis can further integrate with other modules (such as the carbon fixation pathway) to enable plants to synthesize glucose from CO2, whereas the citric acid cycle can couple with other metabolic pathways to generate energy and precursors for biosynthesis (Akram, 2014).

The examples presented above illustrate how the key evolutionary advantages of modularity can be identified in biological systems across different scales and levels of complexity, showing that the general properties of biological modularity go beyond the specificities of a given system realization.

## 5 Discussion

The ubiquity of modular organization across biological scales, from molecular networks to ecosystems, shows the fundamental importance of this organizing principle in the emergence and evolution of complex life forms. As we have shown above, by compartmentalizing biological systems into relatively autonomous, functionally specialized sub-systems, modularity allows for the reuse and recombination of existing modules to support new functions, enhance robustness, enable efficient information processing, and facilitate the evolution of biological complexity.

**FIGURE 1:**
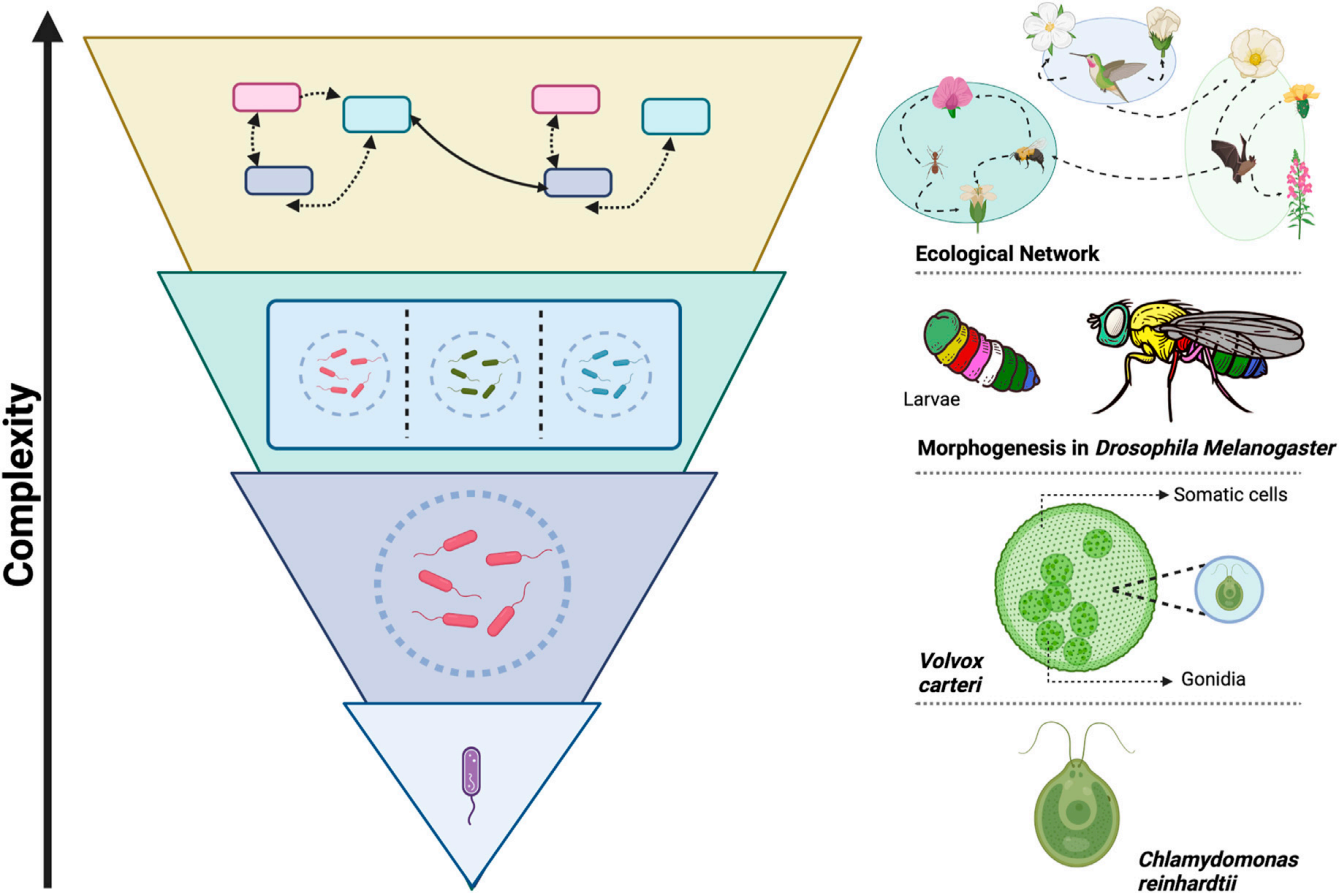
Modularity as a Path to Complexity in Biological Systems. The figure illustrates the role of modularity as a universal organizing principle, observed across multiple scales and biological contexts, that enables the evolution of greater complexity. This complexity arises from the integration of interacting modules, which give rise to new functions and emergent properties at each hierarchical level (Wolf et al., 2018). On the left, a schematic diagram shows how biological systems self-organize modularly at different levels, highlighting their hierarchical nature, where each level is composed of modular subsystems that integrate at higher levels. On the right, specific examples demonstrate this principle across various biological contexts and scales. At the unicellular level, *Chlamydomonas reinhardtii* can form colonies like *Volvox carteri*, an organism in transition towards multicellularity. In these colonies, cells organize into modules specialized in reproduction (gonidia) and motility (somatic cells), improving efficiency and division of labor (Herron, 2016). At the multicellular level, modular organization is observed in various processes, such as morphogenesis in *Drosophila melanogaster*, where Hox genes facilitate the formation of specialized modules and complex structures for diverse physiological functions (Hubert and Wellik, 2023). At the ecosystem level, networks of interactions between species also exhibit modularity, with groups of species interacting more closely with each other, contributing to ecosystem stability and resilience (Olesen et al., 2007). This framework provides an integrative perspective for understanding the role of modularity in the evolution of biological complexity. This image was created with BioRender.com

Understanding modularity as a fundamental principle of organization across scales could unveil its power as a unifying concept, placing it among the few universal principles proposed to explain the remarkable tendency of evolution to generate increasingly complex systems. Figure 1 illustrates this principle, showcasing modularity’s role in biological complexity through specific examples at different levels of organization. Another such principle is criticality, which refers to the state of a system at the boundary between order and chaos, where it exhibits a balance between robustness and adaptability (Munoz, 2018). Robustness refers to a system’s ability to maintain its functionality while facing perturbations, while adaptability refers to its capacity to adjust to changing conditions (Wagner, 2005; Whitacre, 2012). Notably, modularity and criticality share essential features that enhance robustness and adaptability. For example, modularity contributes to robustness by localizing perturbations within modules, and it supports adaptability by enabling the recombination of evolved modules as a faster way to adjust to new conditions, rather than having to develop entirely new solutions (Kashtan and Alon, 2005; Clune et al., 2013).

This striking convergence of modularity and criticality raises thought-provoking questions: Could these principles be deeply interconnected, representing complementary facets of a more fundamental organizational framework? Might the modular architecture of biological systems facilitate their self-organization towards critical states, thereby unlocking the adaptive advantages associated with criticality (Irani and Alderson, 2023)? The intriguing parallels between modularity and criticality invite us to explore the interplay between these properties, potentially uncovering a more comprehensive understanding of the principles that shape the structure and dynamics of complex biological systems across scales.

Despite the significant progress made in understanding the modular organization of biological systems, many challenges and open questions remain. The development of more advanced computational tools for detecting and analyzing modularity across scales could provide deeper insights into the structure and function of complex biological networks. Furthermore, exploring the interplay between modularity and other organizational principles, such as hierarchy and criticality, could provide novel design principles for engineered systems.

The emerging era of cell engineering harnesses the modularity of cells to program complex biological functions, paving the way for transformative advances in biotechnology and medicine (Lim and Pawson, 2010; Lim, 2022). By unraveling the mechanisms that enable the integration of lower-level modules into increasingly complex hierarchies, we may gain a deeper understanding of the processes that gave rise to the first living organisms and the subsequent evolution of biological complexity (Ruiz-Mirazo et al., 2017).

The perspective that we present here highlights the importance of modularity and hierarchical organization as fundamental principles in the design and function of living systems across multiple scales. By identifying the key evolutionary advantages conferred by modular organization, we provide a unifying lens for understanding the emergence of modular hierarchical structures in biology and the mechanisms underlying the resilience, adaptability, and evolvability of living systems. This knowledge not only improves our fundamental understanding of biology but also provides opportunities for applications in a variety of fields, from bioengineering to the design of complex adaptive systems.

## Data Availability

The original contributions presented in the study are included in the article/Supplementary Material, further inquiries can be directed to the corresponding author.
